# Connexin 43, Bcl-2, Bax, Ki67, and E-cadherin patterns in oral squamous cell carcinoma and its relationship with GJA1 rs12197797 C/G

**DOI:** 10.4317/medoral.25298

**Published:** 2022-06-19

**Authors:** Ignacio González Segura, Dante G Secchi, María Fernanda Galíndez, Andrés Carrica, Ronell Bologna-Molina, Mabel Brunotto, Viviana A Centeno

**Affiliations:** 1 DDS. Universidad Nacional de Córdoba, Facultad de Odontología, Departamento de Biología Bucal, Instituto de Investigaciones en Ciencias de la Salud, (INICSA-CONICET-UNC), Ciudad Universitaria, Córdoba, Argentina; 2 PhD. Universidad Nacional de Córdoba, Facultad de Odontología, Departamento de Patología Bucal, Ciudad Universitaria, Córdoba, Argentina; 3 PhD. Molecular Pathology in Stomatology, School of Dentistry, Universidad de la República. Uruguay; 4 ORCID:0000-0001-8010-1079. PhD, MSc. Universidad Nacional de Córdoba, Facultad de Odontología, Departamento de Biología Bucal. Instituto de Investigaciones en Ciencias de la Salud, (INICSA-CONICET-UNC), Ciudad Universitaria, Córdoba, Argentina; 5 ORCID: 0000-0002-2759-115X. PhD. Universidad Nacional de Córdoba, Facultad de Odontología, Departamento de Biología Bucal. Department of Physiology and Cell Biology. University of Arkansas for Medical Sciences (UAMS)

## Abstract

**Background:**

To our knowledge, there is no useful and accurate prognostic biomarker or biomarkers for patients with oral squamous cell carcinoma (OSCC), a tumor with uncertain biological behavior, and unpredicTable clinical progress. The purposes of this study were: a) to determine the expresión profile of Connexin 43, Bcl-2, Bax, E-cadherin, and Ki67 in patients with OSCC; b) identify the GJCA1 rs12197797 genotypic composition.

**Material and Methods:**

A cross-sectional study using genomic DNA and biopsy samples extracted from the oral mucosa with/without OSCC, older than 18 years, both genders, attended at Facultad de Odontología, Universidad Nacional Córdoba. Immunostaining for Cx43, Bcl-2, Bax, E-cadherin, and Ki67 and genotyping GJA1 rs12197797 by RFLP were performed. Odds Ratio (95% CI), Spearman Coefficient were estimated. Mann-Whitney test was applied to analyze immunostaining between controls/cases (*p* <0.05 was set for statistical significance).

**Results:**

GG (mutant) was the most frequent genotype in patients with OSCC diagnosis (53.2%) in relation to CC “healthy” genotype (*p*=0.00487; OR=7.33; CI95% [1.1-54.7]). And, the allele G (mutant) had a presence in 75.5% of OSCC patients. However, no significant association was observed between alleles C/G and diagnosis (*p*=0.0565). The heterozygous genotype was the most frequent in the patients of both groups Cx43 and E-cadherin markers were lower in OSCCs in relation to controls. Ki67 and Bcl-2 immunolabeling were high on OSCC, and Bax immunomarker was diminished in OSCC.

**Conclusions:**

We hypothesized that the oral epithelium losses Connexin 43 and E-cadherin in the membrane, which modifies cell differentiation. The Ki67 and Bcl2 overexpression would increase the cell density in the tissue, by promoting proliferation and decreasing apoptosis. And, this study shows evidence that patients who carry on allele G of GJA1rs12197797 could be at risk of developing OSCC.

** Key words:**Cx43, E-cadherin, Ki67, Bax, Bcl-2, immunostaining expression profile, GJA1 rs12197797 genotyping.

## Introduction

Head and neck cancer (HCC) is the sixth most common cancer in the world, with a high morbidity rate and a low survival rate. 50% of these cancers are of the oral cavity, and oral squamous cell carcinomas (OSCC) are the most frequent (1). Early detection of this pathology enables intervention in the early stages of the disease and reduces the high morbidity of OSCC (2).

The transformation of normal oral epithelium to neoplastic is the result of a series of genetic mutations, which lead to the progressive loss of cell proliferation control mechanisms and cell apoptosis (3). The identification of biological markers helps to improve early diagnosis and the prognosis of malignancy, to monitor the population at risk and prevent recurrence in patients with this pathology and may identify therapeutic targets against metastasis and chemoresistance (4).

Among the potential biomarkers of oral tumorigenesis is Connexin 43 (Cx43), which belongs to the family of proteins that constitute the communicating bonds (GJIC) that facilitate intercellular communication. The altered gene expression of these molecules alters tissue homeostasis, growth, and cell differentiation, facilitating cellular proliferation. Cell proliferation is considered one of the most important biologic mechanisms in tumorigenesis. Ki67 is a proliferative marker, which increases in the S phase and correlates with the cellular growth, making it an excellent marker for this cell process (5). Another biomarker of carcinogenesis is Bcl-2; this family of proteins regulates cell death through mitochondrial integrity and mitochondrial-initiated apoptosis. The family includes antiapoptotic (Bcl-2 and Bcl-xL) and proapoptotic (Bax, Bid, Bak, and Bcl-x) members; balancing their interaction ensures proper apoptotic regulation and cell fate in response to fetal development signals, tissue homeostasis, tumor surveillance, and cell stresses (6).

Molecules that activate the epithelial-mesenchymal transition program (EMT) are involved in the acquisition of the malignant phenotype and subsequent metastasis (7). Intercellular adhesion is maintained mainly by adhesion molecules called cadherins, and specifically by one particular type, E-cadherin, in epithelial tissue, which is a transmembrane glycoprotein and is considered a tumor suppressor gene for negatively regulating cell proliferation and allowing metastasis (8). In OSCC, its low expression is associated with clinical and histopathological characteristics of malignancy, such as metastasis, tumor recurrence, low survival, and altered cell differentiation (9). Analyzing the protein expression of Cx43, Bcl-2/Bax, E-cadherin and Ki67, with potential roles in tumor development, may be associated with the risk of oral cancer malignancy, and is a novel contribution to the knowledge of oral tumorigenesis events and to the selection of new targets for creating prognostic tools for this disease.

Identifying individual genetic predispositions for developing oral cancer is an important strategy in the development of new tools for its prevention and early detection, and for the interpretation of the expression of proteins such as those mentioned above (10).

Latin-American countries like Argentina do not have data on the genetic composition of the population, which all share African, European and Native American genetic ancestry, reflecting a complex demographic history with multiple migrations and mixing events in pre- and post-colonial times (11). Therefore, recognizing the genetic composition of the Córdoba population is important for identifying genotypes of cancer risk. Among the genes encoding these proteins, encoding the gap junction alpha-1 (GJA1) protein has been identified as a tumor suppressor, and the expression of Cx43, produced by this gene, is negatively regulated in many human cancers (12). Some of its polymorphisms, such as that included in this study, have been identified in Latin American populations (13).

For the above reasons and considering that carcinogenesis of the oral epithelium is a multifactorial event, we proposed as objectives: a) to determine the pattern of Cx43, Bcl-2, Bax, E-cadherin and Ki67 biomarkers in patients diagnosed with OSCC; and b) to identify the genotype composition of the SNP (single nucleotide polymorphism) GJCA1, encoding the protein Cx43. These biomarkers and the SNP GJCA1 are associated with the risk of malignancy of oral cancer and are a novel contribution to the knowledge of the events of oral carcinogenesis and to the early detection, treatment and monitoring of this type of complex pathology.

## Material and Methods

- Design

**Figure 1:**
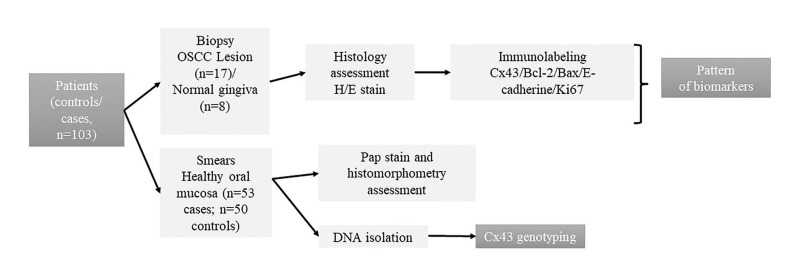
Design chart.

A cross-sectional study was made of oral healthy mucosa DNA (n=82) and biopsy samples (n=25) from patients older than 18 years of both genders, attending the Dentistry School, Córdoba National University, from February 2016 to February 2020. Samples were excluded from patients under corticotherapy or chemotherapy that could modify the symptoms of oral lesions, with systemic diseases, chronic alcoholism, drug use, systemic pathologies, cancers of other locations, and who had previous cancer treatment. Risk habits identified were smoker: current consumption of at least one cigarette⁄ day over a minimum 1-year period; and alcohol: current consumption of 2 drinks⁄ week over a minimum 1-year period. Fig. 1 shows a summary of the study design.

- Immunohistochemistry assessment

The biopsy samples correspond to:

a. Cases (n=17): OSCC diagnosis according to the criteria of the ICD-10 (International Classification of Diseases 10th Revision) of the World Health Organization (https://www.who.int/standards/classifications/classification-of-diseases). Only paraffin-embedded material that met the technical requirements for immunolabeling was included.

b. Controls (n=8): biopsy gingiva samples from patients with indications for tooth extractions by orthodontic reasons and no associated cancer or precancer lesions and clinically healthy, corroborated by Papanicolaou-stained smears (14).

- Connexin 43 (Cx43)

Immunolabeling was performed using the peroxidase-antiperoxidase and avidin-biotin methods, using proprietary protocols (15). Anti-CX43 monoclonal antibody (1:100) was used (Sigma-Aldrich, USA). Negative control was performed by replacing the primary antibody with PBS. The samples were counterstained with Harris hematoxylin. The digital images were obtained with a Motic-BA 400 optical microscope (MOTIC-Asia, Hong Kong), at a final magnification of 400X. The number of positive Cx43 cells was identified from 3 randomly selected fields of 10-3 mm2. The analysis of the immunolabeling of Cx43 was performed through an operator, calibrated using the Software Image Pro Plus v13, and was classified as null, low, medium, and high, according to the criteria of Tanaka *et al* (16).

- Bcl-2 

Immunolabeling was performed using the peroxidase-antiperoxidase and avidin-biotin methods, using manufacturer's protocols. Anti-Bcl-2 monoclonal antibody (1:500) (Cell Marque-SIGMA, USA) was used. Subsequently, CytoScanTM Biotin Detection Kit was used for 10 minutes. The semi-quantitative analysis of Bcl-2 immunoreactivity was performed from 400x digital microphotographs. Positive Bcl-2 cells were counted in the cytoplasm in 3 fields of 10-3 mm2 taken at random. The Bcl-2 immunolabeling was performed by a calibrated operator (Software Image Pro Plus v13). Immunolabeling was classified as null or negative, low, medium, and high, according to the criteria of Tanaka *et al*. (16) and Scherma *et al*. (17).

- Bax, E-cadherin and Ki67

Immunolabeling was performed by the peroxidase-antiperoxidase and avidin-biotin methods, using the manufacturer's protocols. The samples were deparaffinated in the oven at 60ºC for 30 minutes and placed in Xylol for 5 minutes. Antigen recovery was performed with sodium citrate solution 10 mM (high or low pH, depending on each antibody), and microwave heat at 750W in two cycles/5 minutes. Primary antibodies were incubated for 30 minutes, for Bax (Polyclonal, 1:150, Dako Corp, Carpinteria, CA, USA), for Ki-67 (Clone MIB-1, 1:50, Dako Corp, Carpinteria, CA, USA) and E-cadherin (Clone NCH-38, 1:50, Dako Corp., Carpinteria, CA, USA). The number of positive Bax, E-cadherin and Ki67 cells was identified from 3 fields of 10-3 mm2, randomly selected in X400 digital microphotographs. Immunolabeling analysis was performed through a calibrated operator (Software Image Pro Plus v13). Immunoreactivity was considered positive in the cytoplasm for Bax, membrane for E-cadherin, and nuclear for Ki67, and was classified as null, low, medium, and high, according to the criteria of Tanaka *et al* (16) and Scherma *et al*. (17).

- Genotyping GJA1 rs12197797

Buccal cells for DNA extraction were collected from the healthy oral mucosa, corroborated by Papanicolaou stain (14), from controls (n=36) and patients with OSCC (n=46) using disposable sterile cytologic brushes. The cytobrushes were stored in sterile Eppendorf tubes at -30 °C. DNA was isolated as previously described by Galíndez *et al*. 2021 (10). The oral mucosa histomorphometry was performed following own protocols (14)

GJA1 C/G was PCR-amplified using primers 5´-CCTCAGGCTGGTCACACTTA-3´ and 5´-GCTTTTTGCCGTCTATGCAC-3´. PCR products were obtained in a 50 mL final volume. PCR amplification was carried out on an Ivema T18 (Buenos Aires, Argentina) iCycler thermal cycler using the following protocol: 5 min a 95 ºC, 1 min a 95 ºC, 1 min 57 ºC and 1 min 72 ºC for 35 cycles, with an additional 5 min at 72 °C after the last cycle. The PCR products were separated on a 3% TBE (tris/borate/EDTA) agarose gel and stained with GelRed® Nucleic Acid Gel Stain 0.5 mL (Genbiotech, Argentina). A DNA ladder marker (Inbio Highway, Argentina) was used to determine the size of the DNA fragment. The reaction with all reagents excluding the template DNA was used as a negative control. The GJA1 C/G product (110 bp) was digested with Hae III (Thermo Fisher Scientific, Inc) at 37 °C for 3h, and the polymorphisms were determined as CC (110 bp), CG (57, 53, and 110 bp), or GG (53 and 57 bp) (18). The reaction having all the reagents excluding the genomic DNA was used as a negative control.

- Statistical Analysis

The data were described by their absolute and %relative frequencies (qualitative variables) and median/mean/standard error (quantitative variables). The evaluation of the associations between the qualitative variables and the patient's condition was carried out by Irwin-Fisher test. Odds ratios (OR) and their respective 95% confidence intervals (95% CI) were estimated. The correlation of some variables was estimated through the Spearman coefficient (a coefficient ≥0.5 indicates association). The differences between the control and case group percentages (%) of immunostaining were estimated using the Mann-Whitney test. For all tests, *p*<0.05 was set for statistical significance. Data were analyzed by Infostat professional software 2020 (Córdoba, Argentina) and R 4.0.4 (www.r-project.org).

## Results

**Figure 2:**
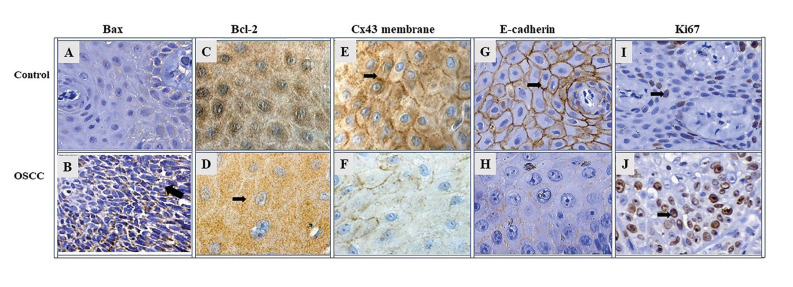
Immunoreactivity photomicrographs at 400x magnification; Bax (A-B); Bcl-2 (C-D); Cx43 membrane (E-F); E-cadherin (G-H); Ki67 (I-J). The number of positive cells from 3 randomly selected 10-3 mm2 fields was identified, obtaining a percentage of cells with: ≤5% (negative); 6 to 25% (low); 26 to 49% (middle); ≥50% (high). Black arrows indicate positive immunostaining.

**Figure 3:**
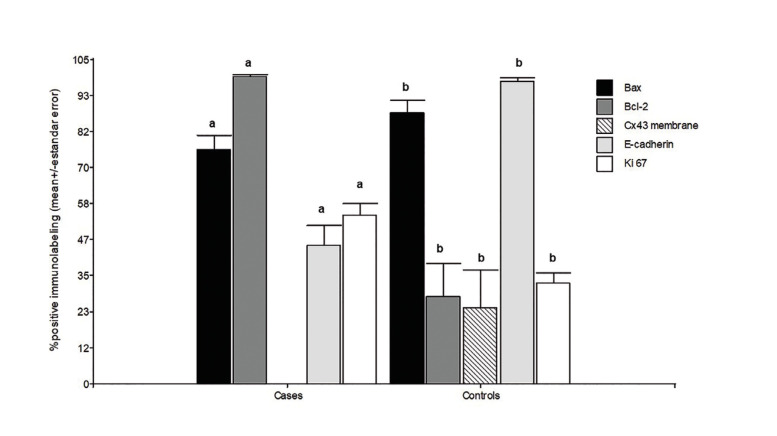
The height of the bars shows the mean values of the percentages of positive immunostaining and the respective standard errors. The total positive values represent the sum of the low, average, and high values of positive immunostaining in each study group. Bax: Control vs Oral Squamous Cell Cancer (OSCC): *p*=0.0302; Bcl-2: Control vs OSCC: *p*=0.0001; Cx43: Control vs OSCC: *p*=0.0001; E-cadherin: Control vs OSCC: *p*=0.0001. Ki67: Control vs OSCC, *p*=0.0007. Mann Whitney test, *p*<0.05 indicates statistical significance.

- Population studied: The DNA samples correspond to a population of 36 controls and 46 cases of oral squamous cell carcinoma (OSCC). From the total population, 25 biopsy samples were recovered. The controls and cases had no statistical differences of age (54.4±16.4 controls vs. 61.03±15.2 cases; *p*=0.1411), but statistical differences were observed in gender (61.29% female / 38.71% male controls vs. 33.33% female / 66.66% male cases; *p*=0.0442; OR=0.32 [0.12-0.86]), tobacco (7.89% yes smoking controls vs. 54. 54% yes smoking cases; *p*=0.0001; OR=14 [3.86-50.81]), and alcohol consumption (13.16% yes alcohol controls vs. 51.51% yes alcohol cases; *p*=0.0008; OR=6.80 [2.20-20.98]).

- Biopsies: 25 samples of patients were recovered and analyzed, with OSCC diagnosis (n=17), and normal gingiva (n=8). 68% of the samples correspond to female and 32% to male patients (*p*=0.0719). The most frequent significant anatomical area of malignant lesion was the tongue (42%), and jugal mucosa (29%). Gingiva (21%), palate (4%), and mouth floor (4%) were other anatomical sites with the presence of malignant lesions (*p*=0.013). The most significant frequent OSCC was the differentiated infiltrating OSCC type (45.83%) (*p*=0.0293).

- Oral mucosa histomorphometry: Nuclear area (AN), cytoplasmic area (AC), and nucleus/cytoplasm ratio (N/C) values were similar to our previous observations corresponding to features of the normal oral mucosa (14).

- Bax: The immunolabeling of Bax was observed on cytoplasm in both groups studied. Bax immunolabeling was significantly higher in middle grade in controls than in cases (Table 1; Fig. 2, Fig. 3).

- Bcl-2: OSCC samples showed a significantly high average percentage of Bcl-2 immunolabeling in comparison with control samples (70% of high grade; *p*=0.0004) (Table 1; Fig. 2, Fig. 3).

- Connexin 43 (Cx43): Cx43 showed remarkable statistically significant immunolabeling in the membrane of control samples (100%, *p*=0.0001), while the OSCC samples showed no Cx43 immunolabeling in their membranes (0%) (Table 1; Fig. 2, Fig. 3).

- E-cadherin: E-cadherin immunostaining was observed at membrane level in all study groups, and a punctate pattern was identified in some samples. E-cadherin immunoreactivity was significantly high in the middle (56.6%) and high (20.7%) grades in control samples compared to OSCC samples (*p*=0.0001) (Table 1; Fig. 2, Fig. 3). Furthermore, a positive correlation was observed between E-cadherin and Connexin 43 (Spearman's coefficient=0.63; *p*=0.0003).

- Ki67: Cells from OSCC samples presented a significantly higher marking (53.1%) for Ki67 than controls (29.8%) (*p*=0.0018) (Table 1; Fig. 2, Fig. 3).

- Gen GJA1: The allelic distribution conforms to the Hardy–Weinberg equilibrium (*p*= 0.4845). The GC genotype was the most frequent in controls (54.1%). Further GG (mutant) was the most frequent genotype in patients with OSCC diagnosis (53.2%) in relation to CC “healthy” genotype (*p*=0.00487; OR=7.33; CI95% [1.1-54.7]). And, the allele G (mutant) had a presence in 75.5% of OSCC patients. However, no significant association was observed between alleles C/G and diagnosis (*p*=0.0565). It was remarkable that the heterozygous genotype was the most frequent in the patients of both groups (Table 2).

**Table 1:**
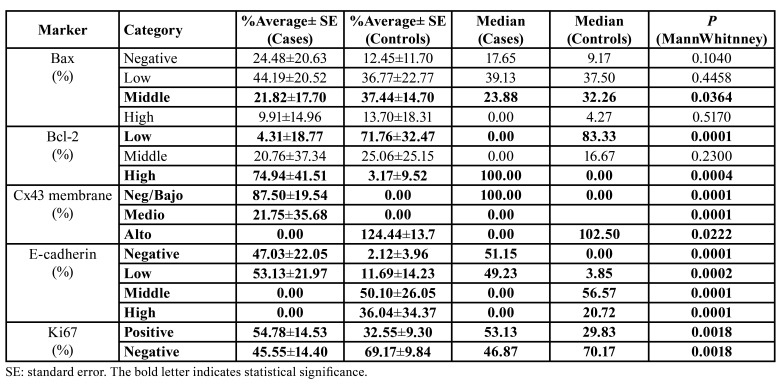
Grade of immunostaining for each biomarker.

**Table 2:**
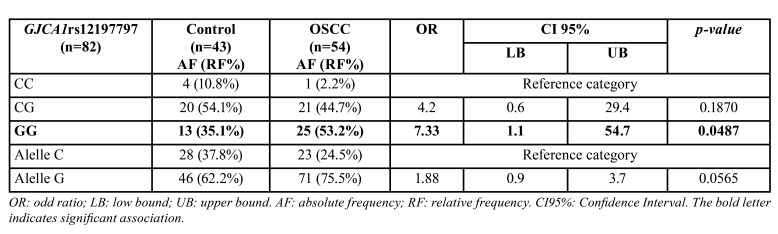
Logic regression model for presence of oral squamous cell carcinoma (OSCC) GJCA1 rs12197797 adjusted by tobacco/alcohol.

## Discussion

Cx43 is one of the potential biomarkers of tumorigenesis in the oral mucosa (18). Our results showed that this protein was not expressed in the membrane of cells from OSCC patient samples. These results match those of Brockmeyer *et al*. in samples of OSCC patients (18). Studies in breast cancer showed that silencing Cx43 expression contributes to breast carcinogenesis, due to enhanced cell proliferation, lost cell polarity, misorientation of the mitotic spindle, and lost multilayer architecture of epithelial tissues. These cellular alterations activate signaling pathways that allow the invasion of non-tumoral tissues (19).

In OSCC biopsy specimens, we reported significant low and null E-cadherin expression relative to controls. Studies have shown that E-cadherin expression in OSCC was associated with clinical and histopathological features of malignancy, such as metastasis, recurrence, poor survival, and poor tumor differentiation (20). There are some reports that analysis of E-cadherin protein expression may be used as a marker of high risk of malignancy (21), for example, for the prognosis of oropharyngeal squamous cell carcinoma (OPSCC) or head neck squamous cell carcinoma (HNSCC). In the latter, E-cadherin expression was associated with an 11.11 lower risk of death from OPSCC (OR=0.09; 95% CI = 0.01-0.88) (21). In contrast, control gingival tissues showed a high and moderate intensity of expression in E-cadherin-positive cells, mainly in the stratum spinosum and granular layer and less in basal cells. In normal mucosa, the high/middle expression of E-cadherin indicates that intercellular adhesion, epithelial structure and function, cell polarity, and regulating intracellular signaling pathways are preserved (9). In addition, we observed an association between E-cadherin and Cx43 (both membrane proteins), with a notably low or null expression in patients with OSCC, coinciding with other studies in other cancers (22).

Our results on Bcl-2 showed increased expression at the cytoplasmic level in OSCC samples relative to that in control tissue. Juneja *et al*. (23) and Coutinho *et al*. (24) report that Bcl-2 family proteins are also present in normal oral mucosa and show lower average expression values than those found in neoplastic cells.

On the other hand, differences were observed in Bax immunoreactivity within OSCC samples of defined tissue architecture without an established cellular organization. In tumors with infiltrating and/or semi-differentiated anatomopathological features, immunolabeling was lower in a number of Bax-positive cells and in the intensity of staining at the cytoplasmic level than in histologically differentiated OSCC. Studies by Zhang *et al*. agree with our findings regarding the higher cytoplasmic expression of Bax in well-differentiated tumors than in poorly differentiated tumors, suggesting that downregulation of Bax in OSCC development promotes a more aggressive phenotype (25). In OSCC, positive cells with increased immunoreactivity were recorded at the periphery of differentiating epithelial tumor islands, with labeling decreasing toward the center of the neoplastic nests; this observation is consistent with the studies performed by Camisasca *et al*. in their OSCC samples (26). In control tissue, the cytoplasmic labeling of Bax-positive cells was mainly on the stratum spinosum and part of the basal layer, losing reactivity towards the surface layers. In turn, in some Bax-positive cells, perinuclear labeling was observed. Bose *et al*. report similar findings in their control samples of the squamous epithelium of the oral cavity, reporting mainly weak expression and nuclear localization (27). A progressive decrease in Bax expression is associated with tumor progression. However, the compensatory increase in its expression may be an early response to oral tumorigenesis (6). The aberrant expression of Bcl-2 and Bax, in cooperation with other molecular changes, suggests a relevant role in OSCC carcinogenesis.

Our OSCC samples reflected an increased number of Ki67-positive cells relative to control tissue. Immunohistochemical staining for Ki67 is widely used as an indicator of proliferation in tumor samples and to analyze its association with prognosis (28). Expression of the nuclear protein Ki67 is strongly correlated with proliferation. Ki67 is expressed in all phases of the cell cycle except G0, making it a neoplastic marker. Ki67 expression is widely used as a marker of proliferation in tumor samples and to analyze its association with prognosis (5).

**Figure 4:**
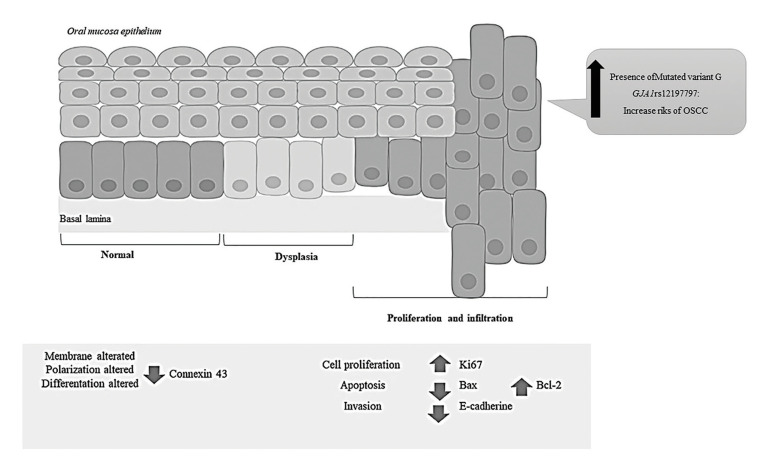
Summary diagram of biomarker alterations in tissue samples from patients with Oral Squamous Cell Cancer (OSCC).

In summary, the study of these markers, associated with different cellular processes, enabled us to establish a pattern of behavior of these markers in patients with OSCC, as shown in Fig. 4. We hypothesized that normal cells receive external stimuli that can damage them, generating genetic changes and leading to cell proliferation and spread. Possibly, the first alteration in the epithelium of the oral mucosa is the loss of Connexin 43 in the membrane, which modifies cell differentiation. The increase in Ki67 (cyclin) and Bcl2 (anti-apoptotic) would than raise the cell density in the tissue by increasing proliferation and decreasing apoptosis, respectively. Another marker that would modify the programmed cell death rate is Bax (pro-apoptotic) because cell apoptosis could be negatively altered. E-cadherin may also be associated with metastasis or act first in conjunction with Connexin 43. Our results show an association between these.

There is no scientific literature on population characterization in Córdoba, Argentina, of the genetic polymorphism evaluated in this study, nor in relation to the variations that can be observed in a population of patients with oral cavity cancer. Our team has already performed genotyping of other polymorphisms in this same population (10), and all our studies are aimed at improving or implementing prevention strategies for this type of cancer. In this research, we observed that 53.2% of the patients diagnosed with OSCC presented the mutated genotype of this polymorphism (GG), suggesting that the mutated variant may alter communication between the cells of the oral mucosal epithelium.

As previously mentioned, mutations in gap junction proteins (connexins), and experimental data using knock-out mice for connexin, have provided significant evidence that gap junction (Gj) intercellular communication is crucial for tissue function and is a major mechanism for tumor cells to communicate with other tumor cells and their surrounding microenvironment to survive and proliferate. Low expression of connexin favors cancer cell proliferation and thus the development of primary tumors [41] In vitro studies in OSCC cell lines would be necessary to study the mechanism underlying the mutant variation of this study.

Gjs could be used to restore the normal conditions of tumor cells or for cancer therapies. The permeability of Cx43 channels to small molecules and macromolecules makes them very attractive targets for delivering drugs directly to the cytoplasm. Cancer cells that overexpress Cx43 may be more permeable and sensitive to chemotherapeutics (29).

On the other hand, the higher frequency of the mutated G allele in the population identifies a population profile different from that described in countries such as Brazil for this SNP (18).

The limitations of the present study include the small number of cases, as well as the retrospective nature of the case-control design, which is prone to selection and information bias (30).

In conclusion, to our knowledge, the present study is the first report of significant association between the G allele of GJA1 rs12197797 with OSCC in the Argentina population. This genetic result together with a pattern of biomarkers could be an aid in the clinical practice to identify patients with risk of OSCCs.
